# A biological invasion reduces rates of cannibalism by Japanese toad tadpoles

**DOI:** 10.1038/s41598-023-36743-8

**Published:** 2023-06-13

**Authors:** Michael R. Crossland, Richard Shine, Takashi Haramura

**Affiliations:** 1 School of Life and Environmental Sciences, University of Sydney, Camperdown, NSW 2006 Australia; 2 School of Natural Sciences, Macquarie University, North Ryde, NSW 2109 Australia; 3 Department of Environmental Sciences, Rakuno Gakuen University, Hokkaido, Japan

**Keywords:** Evolution, Ecology, Evolutionary ecology

## Abstract

Biological invasions can favour rapid changes in intraspecific competitive mechanisms such as cannibalism by imposing novel evolutionary pressures. For example, cane toad (*Rhinella marina*) tadpoles are highly cannibalistic on eggs and hatchlings in their invasive range in Australia, but not in their native range in South America. Whether such changes in cannibalism occur in invasive populations of other amphibian species is unknown. To explore this question, we collected wild-laid egg clutches of Japanese common toads (*Bufo japonicus*) from native and invasive populations in Japan, and conducted laboratory experiments to examine cannibalism responses. Contrary to the Australian system, we found that invasion has been accompanied by reduced cannibalistic tendency of *B. japonicus* tadpoles. This reduction has occurred despite invasive-range *B. japonicus* eggs/hatchlings being more vulnerable than native-range *B. japonicus* eggs/hatchlings to cannibalism by native-range conspecific tadpoles, and to predation by native-range frog tadpoles. Our findings thus support the idea that biological invasions can generate rapid changes in rates of cannibalism, but also show that decreases as well as increases can occur. Future work could investigate the proximate cues and selective forces responsible for this rapid decrease in rates of cannibalism by tadpoles in an invasive *B. japonicus* population.

## Introduction

Biological invasions offer a unique opportunity to examine rapid adaptive changes induced by exposure to new challenges (e.g.^1–4^). A species extending its range into new, unoccupied areas may encounter a suite of novel predators, prey, parasites, competitors and abiotic extremes^5,6^. In response to the opportunities and risks posed by those new interactions, invaders sometimes exhibit remarkably rapid evolved shifts in morphology, physiology, and behaviour^7^.

Some of the best examples of rapidly evolved changes in invasive species come from studies on a South American anuran (the cane toad, *Rhinella marina*) that was introduced to Australia in 1935 in an ill-considered attempt to control insect pests^8^. Although the toad invasion of Australia has been in progress for less than a century, the animals have already expanded their range by thousands of kilometres^9^. In the process of that accelerating range expansion, cane toads have accumulated a wide range of heritable differences in traits that affect locomotor ability and resilience to novel abiotic challenges, both within invasive-range populations (range core vs. range edge) and between native-range *versus* invasive-range populations^10–13^.

One of the most spectacular examples involves the evolution of cannibalism. In the cane toad’s native range in South America, tadpoles rarely consume conspecific eggs or hatchlings^14^. In contrast, tadpoles from all Australian populations tested to date are voracious cannibals, actively searching out and consuming hatchings as they emerge from the egg string^14,15^. This predation is species-specific; cane toad tadpoles ignore the eggs of other anuran species unless they are simultaneously exposed to the chemical cues (bufadienolide toxins) that are present in conspecific eggs and hatchlings^16^. Cannibalism rate is positively correlated with population density in other taxa^17^, and the evolution of intense cannibalism in Australia has been attributed to higher abundances of cane toads in the invasive range than in the native range, placing a selective premium on traits that maximise intraspecific competitive ability^15^. Consistent with that hypothesis, laboratory and outdoor mesocosm experiments show that cannibalism enhances larval viability of invasive cane toads in Australia by reducing competitor abundance^18^.

The greatest challenge to broadly understanding the evolution of cannibalism in invasive amphibian species is the paucity of data. For example, tadpoles of the American bullfrog (*Rana* (*Lithobates*) *catesbeiana*) are known to cannibalise conspecific eggs in the species’ invasive range in China^19^, however, whether this represents altered cannibalism rate post-invasion is unknown due to the lack of comparable cannibalism data from the species’ native range. To our knowledge, only one amphibian species (the cane toad) has been tested for differences in cannibalism in native *versus* invasive populations^14^. To assess the generality of these results, we need to examine other invasive amphibian species. To add to this area of research, we compared rates of cannibalism by a toad species (Japanese common toad, *Bufo japonicus*) that has been translocated from the island of Honshu (native range) to the northern island of Hokkaido (invasive range). The translocation occurred around 100 years ago, similar to the timescale of the invasion of Australia by cane toads^20,21^. Like Australia, Hokkaido had no native bufonid species prior to the invader’s arrival^20,21^. These similarities between the two bufonid species which share life histories^22^, provide a unique opportunity to test whether (1) invasion affects intensity of cannibalism among amphibians in the same ways, and (2) if any such changes are due to evolved shifts in vulnerability of eggs and hatchlings *versus* predatory behaviour of tadpoles.

## Materials and methods

### Study species and area

Japanese common toads lay their eggs in lentic waterbodies and the tadpoles develop in the aquatic environment^23–25^. The shared use of waterbodies as breeding sites with other amphibian species across their range makes *B. japonicus* eggs and hatchlings vulnerable to predation by a variety of anuran tadpoles and salamander larvae^20,24,26–28^. Cannibalism by conspecifics is also possible because, although the breeding period of *B. japonicus* is brief (up to 3 weeks on Honshu^26,27^), eggs hatch after 1 week^20^, giving tadpoles of early clutches a short window of time to prey upon the offspring of later-breeding conspecifics. Hatchlings typically experience high levels of predation pressure by aquatic predators prior to attaining locomotor capacity^20,28^. The eggs and hatchlings of *B. japonicus* contain maternally-invested toxins^29,30^; such toxins often act as a deterrent against aquatic predators^31^. However, amphibians that are native to Honshu and hence, have a long history of sympatry with *B. japonicus*, readily consume invasive-range *B. japonicus* hatchlings and can tolerate those toxins, including predatory frog tadpoles (*Rana ornativentris*) and salamander larvae (*Hynobius nigrescens*)^20,21^.

In contrast, *B. japonicus* is toxic to amphibians within its invasive range, including on Hokkaido, as these species have no evolutionary history of exposure to bufonids^20,21^. On Hokkaido, tadpoles of *Rana pirica* consume invasive *B. japonicus* hatchlings, but are almost always killed by the toxins^20,21,28,30^. Native Hokkaido salamander larvae (*Hynobius retardus*) also consume invasive *B. japonicus* hatchlings, with survival rates varying from 6 to 77% among populations^20,28^. Predation rates by these native amphibian species on Hokkaido can be high. For example, 90% of *H. retardus* larvae that were offered an invasive-range (Hokkaido) *B. japonicus* hatchling consumed the hatchling, whereas 49% of *R. pirica* tadpoles consumed the hatchling^28^.

### Experimental design

We conducted laboratory experiments using wild-collected clutches to investigate predation by tadpoles on eggs and hatchlings for two *B. japonicus* subspecies (*B. j. formosus* and *B. j. japonicus*) and three species of Ranidae (*Rana japonica*, *R. ornativentris*, *R. dybowski*). In the experiments, both *Bufo* tadpoles and *Rana* tadpoles were tested with eggs and hatchlings of *Bufo* and *Rana* species. The two *Bufo* subspecies did not differ significantly in their predatory responses or vulnerability to predation within the native range (see Results). Therefore, we combined data for these two subspecies, and refer to them collectively as *B. japonicus* for brevity.

Three of our study species (*B. japonicus*, *R. japonica*, *R. ornativentris*) are native to the main islands of Honshu, Shikoku, and Kyushu. *Bufo japonicus* (specifically, *B. j. formosus*) has been introduced to Hokkaido as well as to Sadogashima Island and the Izu Islands^32^. *Rana dybowski* is native to Tsushima Island, located between Kyushu and the Korean Peninsula, and is not sympatric with *B. japonicus* (indeed, this island has no native Bufonid species).

### Collection and husbandry of eggs and tadpoles

We hand-collected egg clutches from waterbodies for use in experiments (1 to 3 clutches per species). Egg clutches were identified to species level by one of us (TH) based on experience. Native-range eggs were collected from Honshu (Tochigi Prefecture: *B. japonicus*, *R. japonica*, *R. ornativentris*; Okayama Prefecture: *B. japonicus*, *R. ornativentris*; Wakayama Prefecture: *R. ornativentris*), Kyushu (Miyazaki Prefecture: *B. japonicus*) and Tsushima Island (Nagasaki Prefecture: *R. dybowski*). Invasive-range *B. japonicus* eggs were collected from Hakodate, Sapporo and Tsukigata on Hokkaido. All eggs were collected early on the morning of deposition. Most were collected from separate ponds, although eggs of *B. japonicus* and *R. japonica* were sometimes collected from the same pond. For frogs, we collected entire egg clutches because it was difficult to collect a sub-sample of eggs without damaging the egg mass. For toads, we collected a subsample of eggs because egg strings could be easily broken into segments.

Eggs were transported in plastic containers (14 cm × 18 cm × 8 cm, filled with pond water) by car, train or plane to the laboratory (Seto Marine Research Station of Kyoto University, and Rakuno Gakuen University) where they hatched. Once hatched, tadpoles were reared in 120 L tanks (66 cm × 86 cm × 34 cm; 1 clutch of ~ 50 tadpoles per tank) filled with aged water. Water was changed every 3 days, and tadpoles were fed a diet of algal pellets (Hikari Algal Wafers, Kyorin) ad libitum daily until used in experiments. We used the early-collected clutches to generate tadpoles as predators, and the later-collected clutches to obtain eggs and hatchlings as prey.

### Laboratory experiments

We conducted a series of laboratory experiments to investigate (1) predation among native-range toads and frogs, (2) predation among native-range and invasive-range toads and frogs, and (3) cannibalism among native-range and invasive-range toads. Experiments were conducted using 1000 ml plastic containers filled with 750 ml aged water. In each experiment, a single tadpole (mid-developmental stage: stage 30 to 35^33^) was randomly allocated to either a control container or an egg treatment container. Tadpoles in egg treatment containers were offered either 5 or 10 anuran eggs (depending on availability) that were placed in the centre of containers. Tadpoles in control containers were fed cat food ad libitum daily for sustenance to ensure any tadpole mortality in control containers was not confounded by lack of food. All eggs were added to egg treatment containers within 24 to 36 h after collection, and thus were of comparable developmental stages for all experiments.

We directly observed predation on eggs and hatchlings during experiments, and recorded the number of eggs or hatchlings eaten by each tadpole every 24 h for a period of 72 h (at which time eggs had developed through the hatchling stage and into free-swimming tadpoles and were no longer vulnerable to predation). We recorded water temperature in each container daily using a thermometer (Takara Thermister D619). We also recorded tadpole mortality daily.

### Experiment 1. Predation among native-range toads and frogs

We assessed predation rates by tadpoles on eggs and hatchlings for native-range toad (*Bufo*) and native-range frog (*Rana*) species to determine the role that tadpole phylogeny and egg/hatchling phylogeny play in the outcome of predator–prey interactions within the native range (i.e., predation by Bufonidae tadpoles vs. Ranidae tadpoles, predation on Bufonidae eggs/hatchlings vs. Ranidae eggs/hatchlings). For these experiments, we tested one toad species (*B. j. japonicus* and *B. j. formosus* combined as *B. japonicus*, as described above) and three frog species (*R. japonica*, *R. ornativentris*, *R. dybowski*; Table 1).

**Table 1:** Toad (*Bufo*) and frog (*Rana*) species from native-range populations tested in tadpole vs. egg/hatchling predation experiments.

Tadpole	Egg/hatchling	N
*Bufo japonicus*	*Bufo japonicus*	25
	*Rana japonica*	20
	*Rana ornativentris*	25
*Rana japonica*	*Rana japonica*	5
	*Rana ornativentris*	5
*Rana ornativentris*	*Bufo japonicus*	15
	*Rana japonica*	10
	*Rana ornativentris*	10
*Rana dybowski*	*Bufo japonicus*	10

*N* number of replicates.

### Experiment 2. Predation among native-range and invasive-range toads and frogs

We tested whether the effect of tadpole phylogeny (Bufonidae vs. Ranidae) on predation varies between native *versus* invasive populations of *B. japonicus* eggs/hatchlings. We did this in two ways. First, we combined the data for all native-range tadpoles tested with invasive-range *B. japonicus* eggs/hatchlings (*B. japonicus* tadpoles N = 45, *R. japonica* tadpoles N = 18, *R. ornativentris* tadpoles N = 30) and compared these data to predation by native-range tadpoles on native-range *B. japonicus* eggs/hatchlings (listed in Table 1). Secondly, we specifically compared the predatory responses of tadpoles of two native-range species to native-range *B. japonicus* eggs/hatchlings *versus* invasive-range *B. japonicus* eggs/hatchlings (respectively, *B. japonicus* tadpoles N = 30 vs. 45*, R. ornativentris* tadpoles N = 45 vs. 30).

### Experiment 3. Cannibalism among native-range and invasive-range toads

We investigated whether propensity for cannibalism by *B. japonicus* tadpoles varies with invasion history by comparing consumption of invasive-range *B. japonicus* eggs/hatchlings by native-range *B. japonicus* tadpoles (N = 45) *versus* invasive-range *B. japonicus* tadpoles (N = 30).

### Statistical analyses

#### General methods

We first discuss methods applicable to all statistical analyses that we conducted. Following this, we discuss statistical methods specific to each experiment.

We analysed predation data in R^34^ as a binomial response to treatment (egg/hatchling eaten vs. not eaten) using logistic regression^35^ and quasi-binomial models to account for data over-dispersion (mixed effects models: package MASS:glmmPQL^36^ followed by Anova (package car^37^)). We conducted post-hoc multiple comparisons among treatments using Tukey tests adjusted with the Holm method (package multcomp^38^). Water temperature and time were included as covariates in all models. Because water temperature values are continuous data, we centred these data on mean values for the dataset in question prior to analysis. Container was included as a random effect in all models to account for non-independence of observations of the same container over time.

In some instances, there was zero predation in all replicates within a treatment, resulting in models failing to reach convergence. When this occurred, we assigned a single egg/hatchling to have been eaten in that treatment to obtain a conservative estimate of treatment effect^35^. For models that included an interaction term, we removed the interaction term when it was non-significant and re-ran the model to obtain final estimates. We retained all main effects in the final models, regardless of their statistical significance. Tadpoles that died in egg/hatchling treatment containers (N = 3 toad tadpoles) were not included in statistical analyses assessing predation rate. Specific details for data analysis for each experiment are listed below.

#### Experiment 1. Predation among native-range toads and frogs

We analysed overall effects of phylogeny on predation using fixed effects of tadpole phylogeny (Bufonidae (toad) vs. Ranidae (frog)), egg/hatchling phylogeny (Bufonidae vs. Ranidae) and their interaction (tadpole phylogeny × egg/hatchling phylogeny). We then conducted a multiple comparisons test to identify differences among all four combinations of the tadpole plus egg/hatchling treatments (i.e., toad tadpole plus toad eggs/hatchlings, toad tadpole plus frog eggs/hatchlings, frog tadpole plus toad eggs/hatchlings, frog tadpole plus frog eggs/hatchlings).

#### Experiment 2. Predation among native-range and invasive-range toads and frogs

We combined the data for all native-range tadpoles tested with invasive-range *B. japonicus* eggs/hatchlings and compared these data to predation by native-range tadpoles on native-range *B. japonicus* eggs/hatchlings. For this analysis, we used egg/hatchling population (native-range vs. invasive-range), tadpole phylogeny (Bufonidae vs. Ranidae) and their interaction (egg/hatchling population × tadpole phylogeny) as fixed main effects.

We then conducted a multiple comparison test to assess differences in predation among all four of the tadpole plus egg/hatchling treatments (i.e., native-range toad tadpole plus native-range toad eggs/hatchlings, native-range toad tadpole plus invasive-range toad eggs/hatchlings, native-range frog tadpole plus native-range toad eggs/hatchlings, native-range frog tadpole plus invasive-range toad eggs/hatchlings).

For native-range tadpoles of *B. japonicus* and *R. ornativentris*, we conducted separate analyses to compare predation by these tadpoles on native-range *B. japonicus* eggs/hatchlings *versus* invasive-range *B. japonicus* eggs/hatchlings. These analyses used *B.* *japonicus* egg/hatchling population (native-range vs. invasive-range) as a fixed effect.

#### Experiment 3. Cannibalism among native-range and invasive-range toads

We analysed cannibalism on invasive-range *B. japonicus* eggs/hatchlings by native-range *B. japonicus* tadpoles *versus* invasive-range *B. japonicus* tadpoles using the fixed effect of *B. japonicus* tadpole population (native-range vs. invasive-range).

### Ethics approval

All procedures were approved by Rakuno Gakuen University Animal Care Committee (permit #DH22D8). This study was carried out in compliance with the ARRIVE guidelines, and all methods were carried out in accordance with relevant guidelines and regulations.

## Results

### Comparison of two subspecies of toads from the native range

We tested native-range *B. j. formosus* tadpoles *versus* native-range *B. j. japonicus* tadpoles as predators on eggs/hatchlings of three anuran taxa (*B. j. japonicus*, *R. japonica*, *R. ornativentris*). There was no significant effect of *B. japonicus* subspecies on rate of egg/hatchling consumption by tadpoles (logistic regression: *B. j. japonicus* eggs/hatchlings t = 0.000, df = 13, p = 1.000, *R. japonica* eggs/hatchlings t = − 0.855, df = 22, p = 0.402, *R. ornativentris* eggs/hatchlings t = -1.929, df = 22, p = 0.067). There was similarly no difference in vulnerability to predation for native-range *B. j. formosus* eggs/hatchlings *versus* native-range *B. j. japonicus* eggs/hatchlings: none of these eggs or hatchlings were eaten by any native-range *Bufo* or *Rana* tadpoles. On this basis, we combined data for the two *B. japonicus* subspecies for subsequent analyses and refer to them as *B. japonicus* (identified as native-range vs. invasive-range).

### Effect of covariates on predation by tadpoles on eggs/hatchlings

Time was a significant covariate in most predation models, while the effect of water temperature on consumption of eggs/hatchlings by tadpoles was more variable (Tables 2, 3, 5, 7, 8).

**Table 2:** ANOVA results for effect of tadpole phylogeny (Bufonidae vs. Ranidae) and egg/hatchling phylogeny (Bufonidae vs. Ranidae) on rates of predation for native-range anuran species.

Fixed effect	Chi-square	df	P
Tadpole phylogeny	25.760	1	< 0.0001
Time	5.841	1	0.0157
Water temperature	63.260	1	< 0.0001
Egg/hatchling phylogeny	11.199	1	0.0008
Time	10.566	1	0.0012
Water temperature	36.041	1	< 0.0001

Analyses were conducted using mean water temperature = 18.0 °C.

**Table 3:** ANOVA results for effect of tadpole plus egg/hatchling phylogeny treatment on rates of predation for native-range anuran species.

Fixed effect	Chi-square	df	P
Tadpole plus egg/hatchling phylogeny	46.085	3	< 0.0001
Time	9.515	1	0.0020
Water temperature	27.2266	1	< 0.0001

Treatment combinations were *Bufo* tadpole plus *Bufo* eggs/hatchlings, *Bufo* tadpole plus *Rana* eggs/hatchlings, *Rana* tadpole plus *Bufo* eggs/hatchlings, *Rana* tadpole plus *Rana* eggs/hatchlings. Analyses were conducted using mean water temperature = 18.0 °C.

### Experiment 1. Predation among native-range toads and frogs

**Figure 1:**
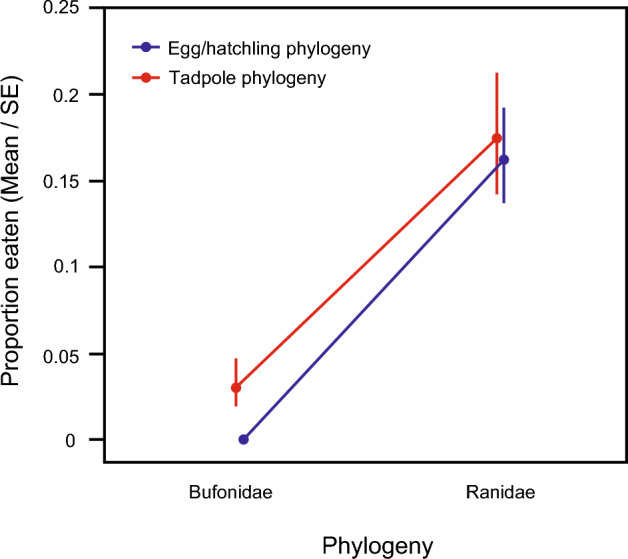
Effect of tadpole phylogeny (Bufonidae vs. Ranidae) and egg/hatchling phylogeny (Bufonidae vs. Ranidae) on rates of predation among native-range amphibian species. Data plotted are number of eggs and hatchlings consumed after 72 h.

Both tadpole phylogeny and egg/hatchling phylogeny were significant predictors of rates of predation for native-range species (Table 2, Fig. 1). Overall, frog tadpoles were more likely to eat eggs/hatchlings (of all kinds tested) than were toad tadpoles (Table 2, Fig. 1), and frog eggs/hatchlings were more likely to be eaten by tadpoles (of all kinds tested) than were toad eggs/hatchlings (Table 2, Fig. 1). The tadpole phylogeny x egg/hatchling phylogeny interaction was non-significant (Chi-square = 2.54, df = 1, p = 0.11).

**Figure 2:**
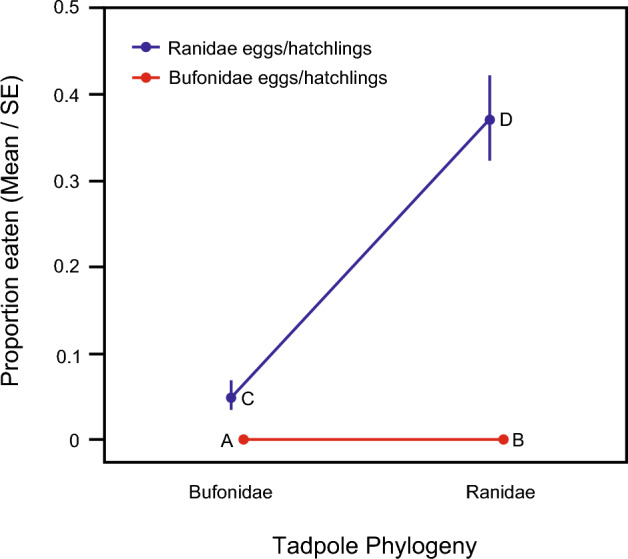
Multiple comparison of tadpole plus egg/hatchling combinations for native-range species. A = toad (*Bufo*) tadpole vs. *Bufo* eggs/hatchlings, B = frog (*Rana*) tadpole vs. *Bufo* eggs/hatchlings, C = *Bufo* tadpole vs. *Rana* eggs/hatchlings, D = *Rana* tadpole vs. *Rana* eggs/hatchlings. Data plotted are number of eggs and hatchlings consumed after 72 h.

Overall, predation varied significantly among the four native-range tadpole plus egg/hatchling treatment groups (Table 3, Fig. 2). No toad or frog tadpoles ate any toad eggs/hatchlings, whereas predation rate by toad tadpoles on frog eggs/hatchlings was low (proportion eggs/hatchlings eaten = 0.05, Fig. 2: respectively A, B, C). The greatest predation rate was by frog tadpoles on frog eggs/hatchlings, with frog tadpoles eating more frog eggs/hatchlings (proportion eaten = 0.37) than toad eggs/hatchlings (proportion eaten = 0.00, Table 4 and Fig. 2: D vs. B). Predation by frog tadpoles on frog eggs/hatchlings was also more common than predation by toad tadpoles on either frog eggs/hatchlings (Table 4 and Fig. 2: D vs. C) or toad eggs/hatchlings (Table 4 and Fig. 2: D vs. A).

**Table 4:** Multiple comparison results for the tadpole plus egg/hatchling treatments in Experiment 1 (native-range species).

Tadpole + egg/hatchling treatment comparison	z	p
*Bufo* tadpole + *Rana* eggs/hatchlings vs. *Bufo* tadpole + *Bufo* eggs/hatchlings; (Fig. 2: C vs A)	1.719	0.2571
*Rana* tadpole + *Bufo* eggs/hatchlings vs. *Bufo* tadpole + *Bufo* eggs/hatchlings; (Fig. 2: B vs A)	0.553	0.6905
*Rana* tadpole + *Rana* eggs/hatchlings vs. *Bufo* tadpole + *Bufo* eggs/hatchlings; (Fig. 2: D vs A)	4.690	< 0.0001
*Rana* tadpole + *Bufo* eggs/hatchlings vs. *Bufo* tadpole + *Rana* eggs/hatchlings; (Fig. 2: B vs C)	− 0.944	0.6905
*Rana* tadpole + *Rana* eggs/hatchlings vs. *Bufo* tadpole + *Rana* eggs/hatchlings; (Fig. 2: D vs C)	5.624	< 0.0001
*Rana* tadpole + *Rana* eggs/hatchlings vs. *Rana* tadpole + *Bufo* eggs/hatchlings; (Fig. 2: D vs B)	3.768	0.0007

Letters in parentheses refer to tadpole plus egg/hatchling treatment combinations identified in Fig. 2. Analyses were conducted using mean water temperature = 18.0 °C.

### Experiment 2. Predation among native-range and invasive-range toads and frogs

**Figure 3:**
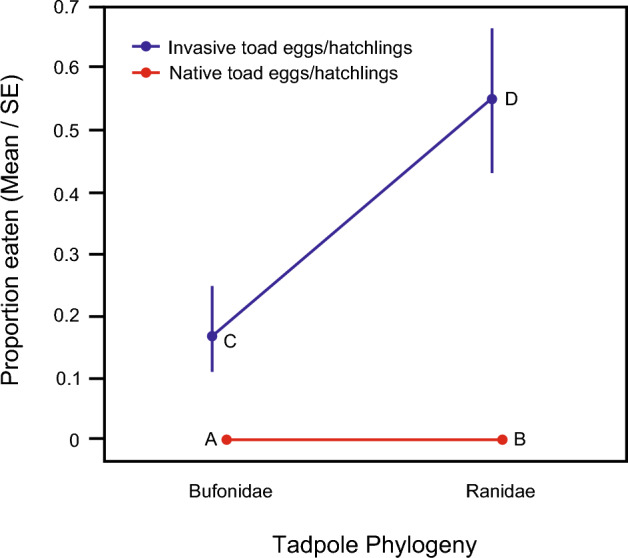
Multiple comparison of tadpole plus egg/hatchling combinations for native-range tadpoles eating native-range toad (*B. japonicus*) eggs/hatchlings (A, B) vs. invasive-range toad (*B. japonicus*) eggs/hatchlings (C, D). Data plotted are number of eggs and hatchlings consumed after 72 h.

Overall, *B. japonicus* egg/hatchling population (native-range vs. invasive-range) was a significant predictor for the rate of predation by native-range tadpoles (Table 5, Fig. 3). Tadpole phylogeny (Bufonidae vs. Ranidae) was also a significant main effect (Table 5). The tadpole phylogeny × egg/hatchling population interaction was non-significant (Chi-square = 2.059, df = 1, p = 0.1513).

**Table 5:** ANOVA results for effect of *B. japonicus* egg/hatchling population (native-range vs. invasive-range) and tadpole phylogeny (Bufonidae vs. Ranidae) on predation by native-range tadpoles.

Fixed effect	Chi-square	df	P
Egg/hatchling population	93.666	1	< 0.0001
Tadpole phylogeny	5.187	1	0.0228
Time	54.763	1	< 0.0001
Water temperature	1.709	1	0.1911

Analyses were conducted using mean water temperature = 20.0 °C.

Specific comparisons among the tadpole plus egg/hatchling treatments showed that tadpole phylogeny was not a significant predictor of predation by native-range tadpoles on native-range *B. japonicus* eggs/hatchlings (Table 6 and Fig. 3: B vs. A) but was a significant predictor for predation by native-range tadpoles on invasive-range *B. japonicus* eggs/hatchlings (Table 6 and Fig. 3: D vs. C). Native-range frog tadpoles ate more invasive-range *B. japonicus* eggs/hatchlings than did native-range toad tadpoles (Table 6 and Fig. 3: D vs. C).

**Table 6:** Multiple comparison results for predation by native-range tadpoles (*Bufo*, *Rana*) on *B. japonicus* (*Bufo*) eggs/hatchlings (native-range vs. invasive-range).

Tadpole + egg/hatchling treatment comparison	z	p
*Bufo* tadpole + native *Bufo* eggs/hatchlings vs. *Bufo* tadpole + invasive *Bufo* eggs/hatchlings; (Fig. 3: A vs C)	− 6.145	< 0.0001
*Rana* tadpole + invasive *Bufo* eggs/hatchlings vs. *Bufo* tadpole + invasive *Bufo* eggs/hatchlings; (Fig. 3: D vs C)	2.733	0.0126
*Rana* tadpole + native *Bufo* eggs/hatchlings vs. *Bufo* tadpole + invasive *Bufo* eggs/hatchlings; (Fig. 3: B vs C)	− 6.209	< 0.0001
*Rana* tadpole + invasive *Bufo* eggs/hatchlings vs. *Bufo* tadpole + native *Bufo* eggs/hatchlings; (Fig. 3: D vs A)	8.030	< 0.0001
*Rana* tadpole + native *Bufo* eggs/hatchlings vs. *Bufo* tadpole + native *Bufo* eggs/hatchlings; (Fig. 3: B vs A)	− 0.113	0.9097
*Rana* tadpole + native *Bufo* eggs/hatchlings vs. *Rana* tadpole + invasive *Bufo* eggs/hatchlings; (Fig. 3: B vs D)	− 8.045	< 0.0001

Letters in parentheses refer to tadpole plus egg/hatchling treatment combinations identified in Fig. 3. Analyses were conducted using mean water temperature = 17.5 °C.

In addition to these phylogenetic effects, both native-range frog tadpoles and native-range toad tadpoles ate more invasive-range *B. japonicus* eggs/hatchlings than native-range *B. japonicus* eggs/hatchlings (Table 6 and Fig. 3: respectively, B vs. D, A vs. C).

**Figure 4:**
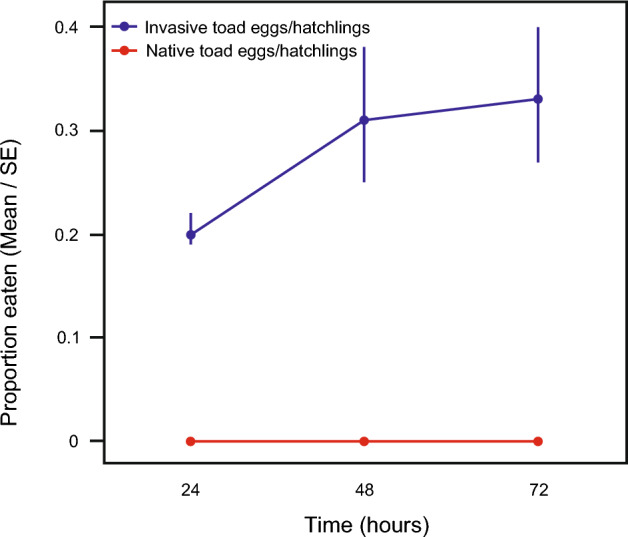
Predation by native-range toad (*B. japonicus*) tadpoles on conspecific native-range eggs/hatchlings vs. conspecific invasive-range eggs/hatchlings. Data plotted are number of eggs or hatchlings consumed at 24 h intervals.

**Figure 5:**
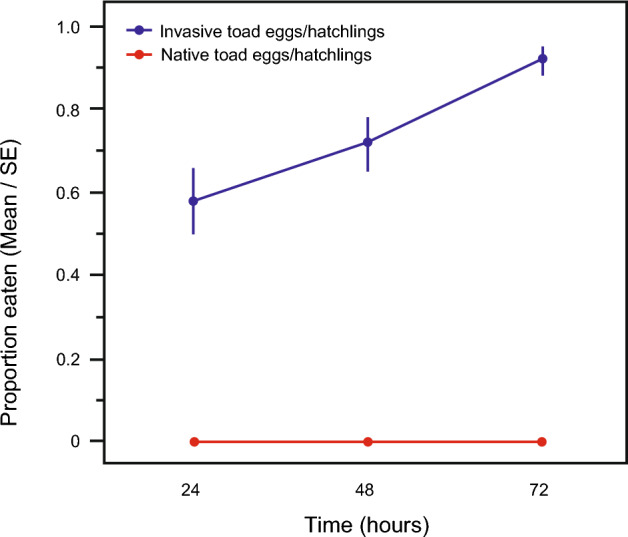
Predation by native-range *R. ornativentris* tadpoles on native-range toad (*B. japonicus*) eggs/hatchlings vs. invasive-range toad (*B. japonicus*) eggs/hatchlings. Data plotted are number of eggs or hatchlings consumed at 24 h intervals.

Individual species comparisons showed that native-range toad tadpoles and frog tadpoles both ate more invasive-range *B. japonicus* eggs/hatchlings than native-range *B. japonicus* eggs/hatchlings (Table 7, Figs. 4 and 5) (Table 7).

**Table 7:** ANOVA results for effect of *B. japonicus* egg/hatchling population (native-range vs. invasive-range) on predation by native-range toad (*B. japonicus*) tadpoles and native-range frog (*R. ornativentris*) tadpoles.

Fixed effect	Chi-square	df	p
*B. japonicus* tadpole			
* B. japonicus* egg/hatchling population	20.288	1	< 0.0001
Time	17.189	1	< 0.0001
Water temperature	1.626	1	0.2022
*R. ornativentris* tadpole			
* B. japonicus* egg/hatchling population	10.619	1	0.0011
Time	3.360	1	0.0668
Water temperature	0.3652	1	0.5456

*B. japonicus* analyses were conducted using mean water temperature = 19.0 °C. *R. ornativentris* analyses were conducted using mean water temperature = 16.8 °C.

### Experiment 3. Cannibalism among native-range and-invasive range toads

**Figure 6:**
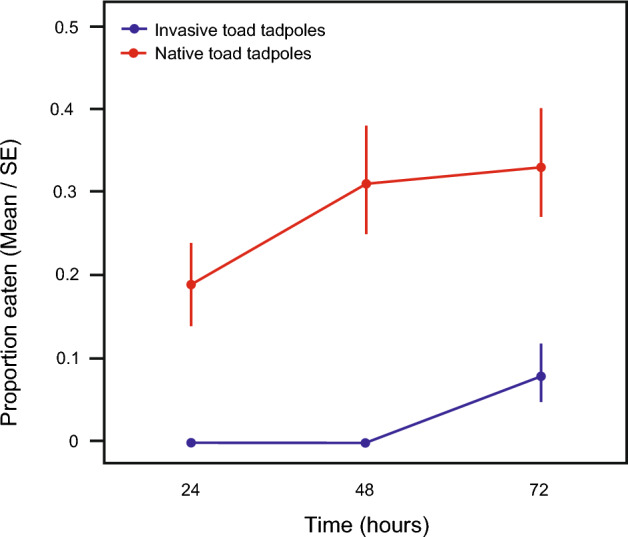
Predation on invasive-range toad (*B. japonicus*) eggs/hatchlings by conspecific native-range tadpoles vs. conspecific invasive-range tadpoles. Data plotted are number of eggs or hatchlings consumed at 24 h intervals.

Native-range *B. japonicus* tadpoles ate more invasive-range *B. japonicus* eggs/hatchlings than did invasive-range *B. japonicus* tadpoles (Table 8, Fig. 6).

**Table 8:** ANOVA results for effect of *B. japonicus* tadpole population (native-range vs. invasive-range) on predation on invasive-range *B. japonicus* eggs/hatchlings.

Fixed effect	Chi-square	df	p
Native-range vs. invasive-range tadpoles	12.643	1	0.0004
Time	30.524	1	< 0.0001
Water temperature	0.010	1	0.9193

Analyses were conducted using mean water temperature = 19.3 °C.

### Tadpole mortality

We did not formally analyse tadpole mortality due to low mortality rates and an obvious lack of treatment effects. During our experiments, 5 *B. japonicus* tadpoles in control containers died, 1 *B. japonicus* tadpole offered frog eggs/hatchlings died without any evidence of predation, and 2 *B. japonicus* tadpoles offered invasive-range *B. japonicus* eggs/hatchlings died after eating either 0 or 1 egg/hatchling.

## Discussion

Within native-range populations, overall rates of predation (both intra- and interspecific) were higher in frogs (Ranidae) than in toads (Bufonidae), demonstrating that frog tadpoles likely play a greater role as predators in structuring native larval anuran communities. For *B. japonicus*, cannibalism was less common in the invasive-range population on Hokkaido than in native-range populations. This post-invasion trend contrasts with another bufonid species (the cane toad) in Australia, and the harlequin ladybird in Europe, both of which exhibit higher rates of cannibalism in invasive populations compared to native populations^14,17^. Although cannibalism may be beneficial for species as they colonise new environments^14,17^, our results demonstrate that invasions do not impact cannibalism responses of all species in the same manner. For *B. japonicus*, invasive populations exist not only on Hokkaido, but also on Sadogashima Island and the Izu Islands^32^. Whether these other invasive *B. japonicus* populations also exhibit reduced propensity for cannibalism is unknown and warrants further investigation.

The lower frequency of cannibalism in the invasive *B. japonicus* population on Hokkaido was due to characteristics of the toad tadpoles and not the toad eggs/hatchlings. Tadpoles of invasive-range *B. japonicus* had a lower propensity for cannibalism (on eggs and hatchlings from the invasive-range population) than did the tadpoles of native-range *B. japonicus*. In contrast, the eggs and hatchlings of invasive-range *B. japonicus* were more prone to being eaten, by both native-range *B. japonicus* tadpoles and native-range frog tadpoles, than eggs and hatchlings of native-range *B. japonicus*. That is, invasive-range *B. japonicus* tadpoles were less likely to exhibit cannibalistic behaviour, but invasive-range *B. japonicus* eggs/hatchlings were more prone to being cannibalised (and eaten by heterospecific tadpoles), compared to native-range *B. japonicus* populations.

We found that native-range tadpoles (*B. japonicus*, *R. japonica*, *R. ornativentris*) consumed eggs and hatchlings of invasive-range *B. japonicus* without ill effect, despite these life history stages possessing toxins^20,21,28–30^. This accords with previous studies that found native-range amphibian species on Honshu (*R. ornativentris* tadpoles, *H. nigrescens* larvae) consume invasive-range *B. japonicus* hatchlings without dying^20,21^. This ability to tolerate *B. japonicus* toxins is likely due to co-evolutionary adaptation on Honshu^20,21^. However, as far as we are aware, our study is the first to examine cannibalism responses on eggs/hatchlings for either native-range or invasive-range *B. japonicus* tadpoles.

Our experimental design does not identify the proximate cues involved in the decreased rates of cannibalism in the invasive population of *B. japonicus* on Hokkaido. Detailed studies in Australian cane toads have shown that toxins released from eggs close to the time of hatching attract cannibalistic tadpoles, and also induce foraging responses by those tadpoles^16,39,40^. Invasive American bullfrog tadpoles in China also are attracted to chemical signals from conspecific eggs, although the precise cue remains unknown^19^. A similar situation to cane toads is plausible with *B. japonicus*, whose eggs and hatchlings also contain toxins^20,21,28–30^. The decrease in rate of cannibalism in the invasive population of *B. japonicus* thus might reflect a lower attraction response by conspecific tadpoles to such cues, or a change in the strength of toxin cues such as a decrease in toxin content, a change in toxin composition, or a decrease in rate of toxin release by eggs/hatchlings. Laboratory experiments exposing predators to specific scent cues rather than entire eggs/hatchlings could test those ideas (cf.^39,40^), and direct measures of the types and amounts of toxins in eggs and hatchlings from each *B. japonicus* population also would be informative (cf.^40,41^). Adult *B. japonicus* exhibit geographic variation in toxin (bufadienolide) composition within their native range^42^. Because the toxins in *B. japonicus* eggs and hatchlings are maternally-invested^29,30^, similar geographic variation presumably also occurs in the toxin composition of *B. japonicus* eggs and hatchlings. However, whether the toxin composition of *B. japonicus* in the invasive range differs from that of native-range populations remains to be determined. Interestingly, the fact that native-range *B. japonicus* tadpoles readily consumed eggs and hatchlings of invasive-range *B. japonicus* suggests that factors other than geographic variation in toxin composition may also be involved in causing lower rates of cannibalism by invasive-range *B. japonicus* tadpoles.

The evolutionary forces responsible for the decline in propensity for cannibalism in invasive *B. japonicus* tadpoles warrant further study, given that invasive cane toad tadpoles in Australian exhibit the opposite pattern^14,15^. The colder climate of Hokkaido (the invasive range) than the native range may affect temporal overlap in contact between conspecific tadpoles and eggs/hatchlings. For example, if breeding is restricted to a brief period in spring, and tadpoles metamorphose during the following summer, then there would be few opportunities for older tadpoles to consume newly-laid eggs and hatchlings. In contrast, a longer breeding season may generate within-year contact between tadpoles and eggs/hatchlings, increasing opportunities for cannibalism. Field studies could clarify that phenology in native *versus* invasive ranges.

The prevalence of cannibalism in populations also covaries with density and the occurrence of other predators^15,43^. For example, the evolution of cannibalism in Australian cane toads has been attributed to higher densities in the invasive range than in the native range, coupled with a scarcity of alternative predators on eggs and hatchlings in the invasive range^14^. If similar selective pressures are responsible for patterns of cannibalism for *B. japonicus* in Japan, we would predict lower densities and/or higher alternate predation pressure in the invasive range of Hokkaido *versus* the native range. To examine these ideas, we need field data on densities of toads in their native range and invasive range, and estimates of rates of egg and hatchling mortality due to intraspecific *versus* interspecific predation. Field enclosures in natural waterbodies may provide a way to measure these variables^14,15^. The availability and nutritional value of alternative food sources for tadpoles also deserve attention, as these factors can influence cannibalism rates^44^.

Although we found a decrease rather than increase in rates of cannibalism in the invasive population of *B. japonicus* on Hokkaido, contrary to the situation with cane toads in Australia^14,15^, it is striking that in both cases a recent invasion (~ 100 years) has initiated a substantial shift in the importance of cannibalism. That similarity supports the idea that invasions impose rapid shifts on the selective forces involved in intraspecific competition generally, and cannibalism specifically. The proximate cues and adaptive significance of such shifts are amenable to studies in the laboratory and in the field, rendering cannibalism in toads an ideal study system in which to examine rapid evolutionary change.

## Data Availability

Data is available from Dryad Digital Repository https://datadryad.org/stash/share/Szhq4YDLypxYit13FGv-J6JQ6LAdtf3lCKsTBp3oZAk.
